# Post Coronavirus Disease 2019 Vaccine-associated Acute Myeloradiculoneuropathy Responsive to Plasmapheresis

**DOI:** 10.1590/0037-8682-0015-2022

**Published:** 2022-06-06

**Authors:** José Wagner Leonel Tavares-Júnior, Pablo Picasso de Araújo Coimbra, Pedro Braga-Neto

**Affiliations:** 1 Universidade Federal do Ceará, Departamento de Medicina Clínica, Fortaleza, CE, Brasil.; 2 Uniclinic Diagnóstico por Imagem - UDI Fortaleza, Centro Universitario Chirstus, Fortaleza, CE, Brasil.; 3 Universidade Estadual do Ceará, Centro de Ciências da Saúde, Fortaleza, CE, Brasil.

**FIGURE 1:**
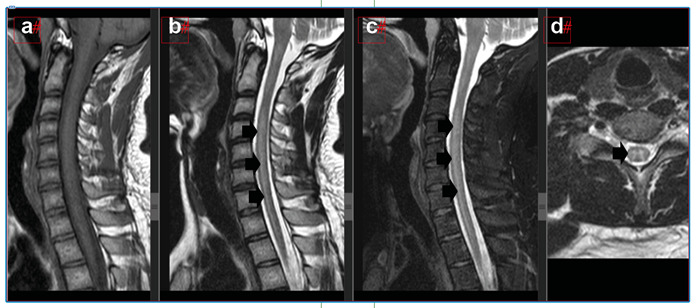
MRI cervical spine in sagittal T1 **(a)**, sagittal T2 **(b)**, sagittal short tau inversion recovery (STIR) **(c)**, and axial T2 **(d)**. Images demonstrate a longitudinally extensive hyperintense signal (**b, c, and d - arrows**). Suggestive of longitudinally extensive transverse myelitis.

A 31-year-old male patient presented with acute tetraparesis and urinary retention 1 day after the first dose of the coronavirus disease 2019 (COVID-19) vaccine. COVID-19 PCR (polymerase chain reaction (PCR) was negative, and IgM (Immunoglobulin M) COVID-19 was reactive. Electromyography revealed an asymmetric motor-sensory axonal polyneuropathy. Cervical magnetic resonance imaging (MRI) revealed longitudinally extensive transverse cervical myelitis (Figure 1). Immunoglobulin treatment for five days improved arm strength. Methylprednisolone was started for five days without improvement. In addition, the patient underwent plasmapheresis with improvement. Blood and cerebrospinal fluid tests were performed, excluding autoimmune diseases, other infections, and neuromyelitis optica (Figure 2). He returned to walking unassisted after 60 days with mild hypoesthesia in his left foot and mild urinary retention. Previous studies reported similar and worse outcomes after the COVID-19 vaccine and other viral infections^1^ ^,^ ^2^. Our report presents one of the earliest cases described after vaccination; however, it has already been registered at a similar time^3^. Such cases usually occur with extended latency periods, probably by severe acute respiratory syndrome coronavirus 2 (SARS-CoV-2) antigens in the COVID-19 vaccine or its chimpanzee adenovirus adjuvant. These antigens can cause myelitis via immune mechanisms^2^. 

**FIGURE 2:** Results of laboratory tests.

Variables	Patient	Reference Values
***Serum tests***		
Hemoglobin (g/dL)	14.7	13-17.5
Leukocytes (cells/mm^3^)	8,100	4,000-11,000
Lymphocytes (cells/mm^3^)	1,701	1,000-3,500
Platelets (number/mm^3^)	253,000	150,000-450,000
C-reactive protein (mg/dL)	>0.6	< 0.6
Aspartate transaminase (U/L)	12	< 37
Alanine transaminase (U/L)	28	< 41
Creatine phosphokinase (U/L)	490	< 190
Anti SSA, Anti SSB antibodies	Negative	Negative
ANCA antibodies	Negative	Negative
Erythrocyte sedimentation rate (mm)	46	0-15
Antiaquaporin-4 antibody	Negative	Negative
Serum Varicella-Zoster IgM	0.65	< 0.8
HIV antibody test	Negative	Negative
Anti-CMV IgM (AU/mL)	0.17	< 1.0
Anti-CMV IgG (AU/mL)	1.10	< 0.5
Anti-HBs hepatitis B (mIU/mL)	< 2.0	< 10.0
Vitamin B12 (pg/mL)	353	211-911
Serum Varicella-Zoster IgG (UI/mL)	567	> 110
VDRL	Non-reactive	Non-reactive
Anti-HCV hepatitis C antibody	Non-reactive	Non-reactive
Antinuclear antibodies	Non-reactive	Non-reactive
Rheumatoid fator	Non-reactive	< 6
Potassium (mmol/L)	4.0	3.5-5.1
Calcium ionized (mg/dL)	8.7	8.6-10.3
Magnesium (mg/dL)	1.8	1.6-2.6
Blood Urea Nitrogen test (mg/dL)	24	15-50
Creatinine (mg/dL)	0.8	0.5-1.3
***Analysis of cerebrospinal fluid***		
Cells (cells/mm^3^)	30	0-4
Cells differential	70% lymphocytes	-
Protein (mg/dL)	61.3	15-45
Glucose (mg/dL)	59	-
VDRL	Not reactive	Not reactive
Gram stain	Negative	Negative
India ink stain	Negative	Negative
Acid-fast stain	Negative	Negative
